# Supplementary material for: Construction of a standardized training system for hospital infection prevention and control for new medical staff in internal medicine ICUs based on the Delphi method
Source: PLoS One. 2023 Nov 16;18(11):e0294606. doi: 10.1371/journal.pone.0294606 (PMC10653407; doi:10.1371/journal.pone.0294606)
Supplement: S1 Data — (ZIP) [file pone.0294606.s001.zip › Data Availability statement/Consultation questionnaire(second round).doc]

**Construction of a competency evaluation index system for front-line nurses during the outbreak of major infectious diseases（the second round）**

Dear expert

Firstly, thank you very much for your guidance and assistance in our project during the first round of inquiry! Based on the valuable suggestions made by experts on various indicators and the statistical results of the first round of correspondence questionnaire, as well as the reference to relevant literature, we combined, deleted, added and modified the indicators at all levels of the first round of correspondence questionnaire with the screening criteria of Arithmetic mean>4.0 items full score>20% and coefficient of variation ≤ 25%, We have formed the second round of inquiry questionnaire (including 4 first level indicators, 28 second level indicators, and 42 third level indicators). We are now sending you the revised second round of inquiry questionnaire. Thank you again for your new opinions and suggestions. Your opinions will provide a basis for establishing the "standardized training mode for hospital infection prevention and control for new medical personnel in the internal medicine ICU"!

This expert inquiry form mainly includes three parts: ① questionnaire on basic information of experts; ② Expert inquiry questionnaire; ③ Expert's judgment basis and familiarity. To ensure research progress, please fill in the questionnaire item by item in order after receiving it. If you have any questions about the questionnaire items, please feel free to contact me at any time. Please rest assured that your personal information and information provided in this study will strictly adhere to the principle of confidentiality. Please reply before June 7th and welcome your valuable feedback! Thank you again for your support and guidance on our project!

Wishing you good health! Work smoothly!

Contacts：Linfei Wu

Phone：15882345312

E-mail：[425515625@qq.com](mailto:425515625@qq.com)

**Part one**

**Basic information questionnaire of experts**

| Name |  | Sex |  | Age |  | Name of hospital |  |
| --- | --- | --- | --- | --- | --- | --- | --- |
| The title of a professional post |  | Position |  | Research field |  | Telephone |  |
| Highest degree | 🞎 pre-university 🞎 university 🞎 post-graduate | | | | | | |
| Years of work in hospital infection prevention and control | 🞎 Years | | | | | | |
| Mentor or not | 🞎 Master's Supervisor 🞎 doctoral supervisor 🞎 No | | | | | | |
| Department |  | | | | | | |

**Part two**

Instructions for filling out the form:

The preliminary inquiry form for the construction of standardized training mode for hospital infection prevention and control for new medical staff in the internal medicine ICU proposed in this study includes 4 first level indicators, 28 second level indicators, and 42 third level indicators. The target audience is: new medical staff in the internal medicine ICU (with a working time of less than 1 year in the ICU).

① Please combine your own experience and understanding to evaluate the items based on the Likert five level scoring method (i.e., 5=very important; 4=important; 3=average; 2=not very important; 1=not important) and the feasibility of the method (i.e., 5=very feasible; 4=feasible; 3=generally feasible; 2=not very feasible; 1=not feasible) (i.e., mark "√" in the corresponding item space, all options are single choice).

② If you believe that the indicator needs to be modified or deleted, please indicate it in the "Modification Opinion" column;

③ If you think there are indicators that need to be supplemented, please fill in the "Suggested Additional Indicators and Their Assignments" column and provide specific assignments. Please choose the most important indicator based on the class hours.

**Table 1 first-level indicators**

| first-level indicators | **Importance score** | | | | | **Expert opinions** |
| --- | --- | --- | --- | --- | --- | --- |
|  | Very important =5 points | More important = 4 points | General =3 points | Not very important = 2 points | Not important = 1 point |  |
| 1 Training content of hospital infection prevention and control |  |  |  |  |  |  |
| 2 Training methods/formats |  |  |  |  |  |  |
| 3 Evaluation content |  |  |  |  |  |  |
| 4 Evaluation Indicators |  |  |  |  |  |  |
| Proposed additional indicators and their significance: | | | | | | |

**Table 2 second-level indicators**

| first-level indicators | second-level indicators | Importance score | | | | | Method feasibility | | | | | **Expert opinions** |
| --- | --- | --- | --- | --- | --- | --- | --- | --- | --- | --- | --- | --- |
|  |  | 5 | 4 | 3 | 2 | 1 | 5 | 4 | 3 | 2 | 1 |  |
| 1 Training content of hospital infection prevention and control | 1.1 Laws and Regulations on Nosocomial Infection |  |  |  |  |  |  |  |  |  |  |  |
|  | 1.2 Nosocomial infection monitoring |  |  |  |  |  |  |  |  |  |  |  |
|  | 1.3 Disinfection and sterilization |  |  |  |  |  |  |  |  |  |  |  |
|  | 1.4 Hand hygiene |  |  |  |  |  |  |  |  |  |  |  |
|  | 1.5 Prevention and management of multidrug-resistant bacteria |  |  |  |  |  |  |  |  |  |  |  |
|  | 1.6 Classification management of medical waste |  |  |  |  |  |  |  |  |  |  |  |
|  | 1.7 Occupational protection |  |  |  |  |  |  |  |  |  |  |  |
|  | 1.8 Ventilator-associated pneumonia prevention and control |  |  |  |  |  |  |  |  |  |  |  |
|  | 1.9 Prevention and control of catheter-related bloodstream infection |  |  |  |  |  |  |  |  |  |  |  |
|  | 1.10 Prevention and control of catheter-related urinary tract infections |  |  |  |  |  |  |  |  |  |  |  |
|  | 1.11 Infectious disease knowledge |  |  |  |  |  |  |  |  |  |  |  |
|  | 1.12 Aseptic technique (Operation) |  |  |  |  |  |  |  |  |  |  |  |
|  | 1.13 Cleaning and Disinfection (Operation) |  |  |  |  |  |  |  |  |  |  |  |
|  | 1.14 Standard Prevention Related Skills (Operations) |  |  |  |  |  |  |  |  |  |  |  |
|  | 1.15 Specimen Collection (Operation) |  |  |  |  |  |  |  |  |  |  |  |
| 2 Training methods/formats | 2.1 Theory lecture + operation demonstration or video |  |  |  |  |  |  |  |  |  |  |  |
|  | 2.2 Clinical case discussions or scenario presentations |  |  |  |  |  |  |  |  |  |  |  |
|  | 2.3 Flipped classroom |  |  |  |  |  |  |  |  |  |  |  |
| 3 Evaluation content | 3.1 Theoretical knowledge examination |  |  |  |  |  |  |  |  |  |  |  |
|  | 3.2 Operational skills examination |  |  |  |  |  |  |  |  |  |  |  |
| 4 Evaluation Indicators | 4.1Qualification rate of knowledge of hospital infection prevention and control |  |  |  |  |  |  |  |  |  |  |  |
|  | 4.2Hand hygiene compliance and correctness rate |  |  |  |  |  |  |  |  |  |  |  |
|  | 4.3Qualified rate of aseptic operation |  |  |  |  |  |  |  |  |  |  |  |
|  | 4.4Correct implementation rate of occupational protection |  |  |  |  |  |  |  |  |  |  |  |
|  | 4.5Number of non-compliant operations |  |  |  |  |  |  |  |  |  |  |  |
|  | 4.6 Incidence of occupational exposure |  |  |  |  |  |  |  |  |  |  |  |
|  | 4.7 Course Training Satisfaction |  |  |  |  |  |  |  |  |  |  |  |
|  | 4.8Incidence of triductal infections |  |  |  |  |  |  |  |  |  |  |  |

**Table 3 third-level indicators**

| first-level indicators | second-level indicators | third-level indicators | Importance score | | | | | Method feasibility | | | | | Expert opinions |
| --- | --- | --- | --- | --- | --- | --- | --- | --- | --- | --- | --- | --- | --- |
|  |  |  | 5 | 4 | 3 | 2 | 1 | 5 | 4 | 3 | 2 | 1 |  |
| 1 Training content of hospital infection prevention and control | 1.1 Laws and Regulations on Nosocomial Infection | 1.1.1“Law of the People’s Republic of China on the Prevention and Treatment of Infectious Diseases” |  |  |  |  |  |  |  |  |  |  |  |
|  |  | “Regulations on Emergency Response to Public Health Emergencies” |  |  |  |  |  |  |  |  |  |  |  |
|  | 1.2 Nosocomial infection monitoring | 1.2.1 Definition of nosocomial infection and reporting requirements |  |  |  |  |  |  |  |  |  |  |  |
|  |  | 1.2.2 Judgment of common nosocomial infections |  |  |  |  |  |  |  |  |  |  |  |
|  |  | 1.2.3 Nosocomial Infection Outbreak Definition and Reporting and Handling Process |  |  |  |  |  |  |  |  |  |  |  |
|  | 1.3 Disinfection and sterilization | 1.3.1 Basic principles of disinfection and sterilization |  |  |  |  |  |  |  |  |  |  |  |
|  |  | 1.3.2 Item classification and selection principles for disinfection and sterilization |  |  |  |  |  |  |  |  |  |  |  |
|  | 1.4 Hand hygiene | 1.4.1 Definition of hand hygiene |  |  |  |  |  |  |  |  |  |  |  |
|  |  | 1.4.2 Indications for hand hygiene |  |  |  |  |  |  |  |  |  |  |  |
|  |  | 1.4.3 Methods and precautions for hand hygiene |  |  |  |  |  |  |  |  |  |  |  |
|  | 1.5 Prevention and management of multidrug-resistant bacteria | 1.5.1 Definition of multi-drug resistant bacteria |  |  |  |  |  |  |  |  |  |  |  |
|  |  | 1.5.2 Key surveillance species of multidrug-resistant bacteria |  |  |  |  |  |  |  |  |  |  |  |
|  |  | 1.5.3 Judgment criteria for multidrug-resistant bacteria |  |  |  |  |  |  |  |  |  |  |  |
|  |  | 1.5.4 Prevention and control of multidrug-resistant bacteria |  |  |  |  |  |  |  |  |  |  |  |
|  | 1.6 Classification management of medical waste | 1.6.1 Definition and classification of medical waste |  |  |  |  |  |  |  |  |  |  |  |
|  |  | 1.6.2 Requirements for Medical Waste Management |  |  |  |  |  |  |  |  |  |  |  |
|  | 1.7 Occupational protection | 1.7.1 Definition of Standard Precautions |  |  |  |  |  |  |  |  |  |  |  |
|  |  | 1.7.2 Selection and use of protective equipment |  |  |  |  |  |  |  |  |  |  |  |
|  |  | 1.7.3 Handling of occupational exposures |  |  |  |  |  |  |  |  |  |  |  |
|  |  | 1.7.4 Safe injection |  |  |  |  |  |  |  |  |  |  |  |
|  | 1.8 Ventilator-associated pneumonia prevention and control | 1.8.1 Definition of Ventilator-Associated Pneumonia |  |  |  |  |  |  |  |  |  |  |  |
|  |  | 1.8.2 Diagnosis of ventilator-associated pneumonia |  |  |  |  |  |  |  |  |  |  |  |
|  |  | 1.8.3 Prevention and Control Strategies of Ventilator-Associated Pneumonia |  |  |  |  |  |  |  |  |  |  |  |
|  | 1.9 Prevention and control of catheter-related bloodstream infection | 1.9.1 Definition of catheter-related bloodstream infection |  |  |  |  |  |  |  |  |  |  |  |
|  |  | 1.9.2 Diagnosis of catheter-related bloodstream infection |  |  |  |  |  |  |  |  |  |  |  |
|  |  | 1.9.3 Prevention and control strategies for catheter-related bloodstream infections |  |  |  |  |  |  |  |  |  |  |  |
|  | 1.10 Prevention and control of catheter-related urinary tract infections | 1.10.1 Definition of catheter-related urinary tract infection |  |  |  |  |  |  |  |  |  |  |  |
|  |  | 1.10.2 Diagnosis of catheter-related urinary tract infection |  |  |  |  |  |  |  |  |  |  |  |
|  |  | 1.10.3 Prevention and control strategies for catheter-related urinary tract infections |  |  |  |  |  |  |  |  |  |  |  |
|  | 1.13 Cleaning and Disinfection (Operation) | 1.13.1 Disinfection of instruments and equipment |  |  |  |  |  |  |  |  |  |  |  |
|  |  | 1.13.2 Terminal disinfection of bed units |  |  |  |  |  |  |  |  |  |  |  |
|  | 1.14 Standard Prevention Related Skills (Operations) | 1.14.1 hand hygiene |  |  |  |  |  |  |  |  |  |  |  |
|  |  | 1.14.2 Correct putting on and taking off of gloves, masks, isolation gowns, protective clothing and other protective equipment |  |  |  |  |  |  |  |  |  |  |  |
|  | 1.15 Specimen Collection (Operation) | 1.15.1 Collection of blood samples |  |  |  |  |  |  |  |  |  |  |  |
|  |  | 1.15.2 Sputum specimen collection |  |  |  |  |  |  |  |  |  |  |  |
|  |  | 1.15.3 Urine specimen collection |  |  |  |  |  |  |  |  |  |  |  |
| 3 Evaluation content | 3.2 Operational skills examination | 3.2.1 Hand hygiene |  |  |  |  |  |  |  |  |  |  |  |
|  |  | 3.2.2 Putting on and taking off masks and gloves |  |  |  |  |  |  |  |  |  |  |  |
|  |  | 3.2.3 Putting on and taking off the gown |  |  |  |  |  |  |  |  |  |  |  |
|  |  | 3.2.4 Collection of blood samples |  |  |  |  |  |  |  |  |  |  |  |
|  |  | 3.2.5 Sputum specimen collection |  |  |  |  |  |  |  |  |  |  |  |
|  |  | 3.2.6 Urine specimen collection |  |  |  |  |  |  |  |  |  |  |  |

**Part three**

**Instructions for completing the form:**

**Please select the basis for your judgement, level of influence and familiarity with the above entry “√” the appropriate column**

| Basis of judgement | Level of influence | | | | |
| --- | --- | --- | --- | --- | --- |
|  | large | middle | | small | |
| Theoretical analysis |  |  | |  | |
| Practical experience |  |  | |  | |
| References |  |  | |  | |
| Intuitive aspects |  |  | |  | |
| How familiar are you with the content of this survey | | | | | |
| Degree of familiarity | Very familiar | More familiar | General familiar | Not really familiar | Completely unfamiliar |
|  |  |  |  |  |  |

Thank you again for your support and help with this subject.I wish you a happy life. Good luck with your work.
