# Supplementary material for: Construction of a standardized training system for hospital infection prevention and control for new medical staff in internal medicine ICUs based on the Delphi method
Source: PLoS One. 2023 Nov 16;18(11):e0294606. doi: 10.1371/journal.pone.0294606 (PMC10653407; doi:10.1371/journal.pone.0294606)
Supplement: S1 Data — (ZIP) [file pone.0294606.s001.zip › Data Availability statement/Consultation questionnaire(first round∩╝ë.doc]

**“Construction of a standardized training system for hospital infection prevention and control for new medical staff in internal medicine ICUs”**

**Consultation questionnaire (first round)**

Dear expert

Thank you for your busy schedule to participate in this research. I am Linfei Wu, a sense control nurse in the West China Hospital of Sichuan University, and I am conducting the research on "the construction of standardized training mode for hospital sense prevention and control of new medical staff in internal medicine ICU". You have been engaged in research related to hospital infection management for a long time, with profound knowledge and rich experience. We cordially invite you as a consulting expert for this study, and kindly request your guidance and valuable opinions. We express our sincerest gratitude for your hard work.

The construction of standardized training mode for hospital infection prevention and control for new medical staff in the internal medicine ICU is based on the knowledge, belief, and behavior model. After systematic literature review and population survey, the data results are sorted out, and finally determined after discussion by the project team. The expert inquiry form mainly consists of three parts: the first part is a questionnaire on the basic information of experts; The second part is the expert inquiry questionnaire. Among them, the expert inquiry questionnaire includes 6 first level indicators, 31 second level indicators, and 41 third level indicators. To ensure the timeliness and accuracy of expert consultation, please fill in the questionnaire item by item in order after receiving it. If you have any questions about the questionnaire items, please feel free to contact me at any time. Please rest assured that your personal information and information provided in this study will strictly adhere to the principle of confidentiality. Please reply before April 27th and welcome your valuable feedback! Thank you again for your support and guidance on our project!

Wishing you good health! Work smoothly!

Contacts：Linfei Wu

Phone：15882345312

E-mail：[425515625@qq.com](mailto:425515625@qq.com)

**Part one**

**Basic information questionnaire of experts**

| Name |  | Sex |  | Age |  | Name of hospital |  |
| --- | --- | --- | --- | --- | --- | --- | --- |
| The title of a professional post |  | Position |  | Research field |  | Telephone |  |
| Highest degree | 🞎 pre-university 🞎 university 🞎 post-graduate | | | | | | |
| Years of work in hospital infection prevention and control | 🞎 Years | | | | | | |
| Mentor or not | 🞎 Master's Supervisor 🞎 doctoral supervisor 🞎 No | | | | | | |
| Department |  | | | | | | |

**Part two**

Instructions for filling out the form:

The preliminary inquiry form for the construction of standardized training mode for hospital infection prevention and control for new medical staff in the internal medicine ICU proposed in this study includes 6 first level indicators, 31 second level indicators, and 41 third level indicators. The target audience is: new medical staff in the internal medicine ICU (with a working time of less than 1 year in the ICU).

① Please combine your own experience and understanding to evaluate the items based on the Likert five level scoring method (i.e., 5=very important; 4=important; 3=average; 2=not very important; 1=not important) and the feasibility of the method (i.e., mark "√" in the corresponding item space, all options are single choice).

② If you believe that the indicator needs to be modified or deleted, please indicate it in the "Modification Opinion" column;

③ If you think there are indicators that need to be supplemented, please fill in the "Suggested Additional Indicators and Their Assignments" column and provide specific assignments. Please choose the most important indicator based on the class hours.

**Table 1 first-level indicators**

| first-level indicators | Importance score | | | | | Expert opinions |
| --- | --- | --- | --- | --- | --- | --- |
|  | Very important =5 points | More important = 4 points | General =3 points | Not very important = 2 points | Not important = 1 point |  |
| 1 Training content of hospital infection prevention and control |  |  |  |  |  |  |
| 2 Hospital infection prevention and control skills training |  |  |  |  |  |  |
| 3 Training methods/formats |  |  |  |  |  |  |
| 4 Training time |  |  |  |  |  |  |
| 5 Assessment indicators |  |  |  |  |  |  |
| 6 Evaluation Indicators |  |  |  |  |  |  |
| Proposed additional indicators and their significance: | | | | | | |

**Table 2 second-level indicators**

| first-level indicators | second-level indicators | Importance score | | | | | Expert opinions |
| --- | --- | --- | --- | --- | --- | --- | --- |
|  |  | Very important =5 points | More important = 4 points | General =3 points | Not very important = 2 points | Not important = 1 point |  |
| 1 Training content of hospital infection prevention and control | 1.1 Laws and Regulations on Nosocomial Infection |  |  |  |  |  |  |
|  | 1.2 Nosocomial infection monitoring |  |  |  |  |  |  |
|  | 1.3 Disinfection and sterilization |  |  |  |  |  |  |
|  | 1.4 Hand hygiene |  |  |  |  |  |  |
|  | 1.5 management of multidrug-resistant bacteria |  |  |  |  |  |  |
|  | 1.6 Classification management of medical waste |  |  |  |  |  |  |
|  | 1.7 Occupational protection |  |  |  |  |  |  |
|  | 1.8 Ventilator-associated pneumonia prevention and control |  |  |  |  |  |  |
|  | 1.9 Prevention and control of catheter-related bloodstream infection |  |  |  |  |  |  |
|  | 1.10 Prevention and control of catheter-related urinary tract infections |  |  |  |  |  |  |
| 2 Hospital infection prevention and control skills training | 2.1 Disinfection and isolation technology |  |  |  |  |  |  |
|  | 2.2 Standard Prevention Related Skills |  |  |  |  |  |  |
|  | 2.3 Specimen Collection |  |  |  |  |  |  |
| 3 Training methods/formats | 3.1 Offline lectures |  |  |  |  |  |  |
|  | 3.2 Online lectures |  |  |  |  |  |  |
|  | 3.3 Online lectures+offline lectures |  |  |  |  |  |  |
|  | 3.4 Theory lecture + operation demonstration or video |  |  |  |  |  |  |
|  | 3.5 Case sharing or scenario demonstration |  |  |  |  |  |  |
| 4 Training time | 4.1 All course content will be completed in one day |  |  |  |  |  |  |
|  | 4.2 The course content is scattered and completed in one week |  |  |  |  |  |  |
| 5 Assessment indicators | 5.1 Theoretical knowledge assessment |  |  |  |  |  |  |
|  | 5.2 Operational skill assessment |  |  |  |  |  |  |
| 6 Evaluation Indicators | 6.1 Compliance and accuracy of hand hygiene |  |  |  |  |  |  |
|  | 6.2 Correctness and compliance of protective equipment use |  |  |  |  |  |  |
|  | 6.3 Course Training Satisfaction |  |  |  |  |  |  |
|  | 6.4 Nosocomial infection rate |  |  |  |  |  |  |
| Proposed additional indicators and their significance: | | | | | | | |

**Table 3 third-level indicators**

| first-level indicators | second-level indicators | third-level indicators | Importance score | | | | | Expert opinions |  |
| --- | --- | --- | --- | --- | --- | --- | --- | --- | --- |
|  |  |  | Very important =5 points | More important = 4 points | General =3 points | Not very important = 2 points | Not important = 1 point |  | |
| 1 Training content of hospital infection prevention and control | 1.1 Laws and Regulations on Nosocomial Infection | 1.1.1“Law of the People’s Republic of China on the Prevention and Treatment of Infectious Diseases” |  |  |  |  |  |  | |
|  |  | 1.1.2Regulations on the Management of Medical Waste and Related Supporting Documents |  |  |  |  |  |  | |
|  |  | 1.1.3Regulations on the Management of Medical Institutions and Their Implementation Rules |  |  |  |  |  |  | |
|  |  | 1.1.4Management Measures for Hospital Infection |  |  |  |  |  |  | |
|  |  | 1.1.5Disinfection Management Measures |  |  |  |  |  |  | |
|  | Nosocomial infection monitoring | 1.2.1 Hospital infection definition and reporting requirements |  |  |  |  |  |  | |
|  |  | 1.2.2 Judgment of common nosocomial infection |  |  |  |  |  |  | |
|  |  | 1.2.3 Definition and reporting process of hospital infection outbreak |  |  |  |  |  |  | |
|  | 1.3 Disinfection and sterilization | 1.3.1 Basic principles of disinfection and sterilization |  |  |  |  |  |  | |
|  |  | 1.3.2 Classification of items and selection principles for disinfection and sterilization |  |  |  |  |  |  | |
|  | 1.4 Hand hygiene | 1.4.1 Definition of Hand Hygiene |  |  |  |  |  |  | |
|  |  | 1.4.2 Indications for hand hygiene |  |  |  |  |  |  | |
|  |  | 1.4.3 Methods and precautions for hand hygiene |  |  |  |  |  |  | |
|  | 1.5 management of multidrug-resistant bacteria | 1.5.1 Definition of multidrug-resistant bacteria |  |  |  |  |  |  | |
|  |  | 1.5.2 Key monitoring types of multidrug-resistant bacteria |  |  |  |  |  |  | |
|  |  | 1.5.3 Criteria for determining multidrug-resistant bacteria |  |  |  |  |  |  | |
|  |  | 1.5.4 Management system for multidrug-resistant bacteria |  |  |  |  |  |  | |
|  | 1.6 Classification management of medical waste | 1.6.1 Definition and classification of medical waste |  |  |  |  |  |  | |
|  |  | 1.6.2 Medical waste management requirements |  |  |  |  |  |  | |
|  | 1.7 Occupational protection | 1.7.1 Definition of standard prevention |  |  |  |  |  |  | |
|  |  | 1.7.2 Correct use of protective equipment |  |  |  |  |  |  | |
|  |  | 1.7.3 Handling of occupational exposure |  |  |  |  |  |  | |
|  |  | 1.7.4 safety injection |  |  |  |  |  |  | |
|  | 1.8 Ventilator-associated pneumonia prevention and control | 1.8.1 Definition of ventilator-associated pneumonia |  |  |  |  |  |  | |
|  |  | 1.8.2 Diagnosis of ventilator-associated pneumonia |  |  |  |  |  |  | |
|  |  | 1.8.3 Prevention and control strategies for ventilator-associated pneumonia |  |  |  |  |  |  | |
|  | 1.9 Prevention and control of catheter-related bloodstream infection | 1.9.1 Definition of catheter-related bloodstream infections |  |  |  |  |  |  | |
|  |  | 1.9.2 Diagnosis of catheter-related bloodstream infections |  |  |  |  |  |  | |
|  |  | 1.9.3 Prevention and control strategies for catheter-related bloodstream infections |  |  |  |  |  |  | |
|  | 1.10 Prevention and control of catheter-related | 1.10.1 Definition of Foley catheter related urinary tract infection |  |  |  |  |  |  | |
|  |  | 1.10.2 Diagnosis of Foley catheter related urinary tract infection |  |  |  |  |  |  | |
|  |  | 1.10.3 Prevention and control strategy of Foley catheter related urinary tract infection |  |  |  |  |  |  | |
| 2 Hospital infection prevention and control skills training | 2.1 Disinfection and isolation technology | 2.1.1 Hand hygiene |  |  |  |  |  |  | |
|  |  | 2.1.2 Aseptic technique |  |  |  |  |  |  | |
|  |  | 2.1.3 Correct putting on and taking off of gloves, masks, isolation gowns, protective clothing and other protective equipment |  |  |  |  |  |  | |
|  |  | 2.1.4 Safe injection and sharp tool handling |  |  |  |  |  |  | |
| 5 Assessment indicators | 5.2 Operational skill assessment | 5.2.1 Hand hygiene |  |  |  |  |  |  | |
|  |  | 5.2.2 Wearing and taking off isolation clothes |  |  |  |  |  |  | |
|  |  | 5.2.3 Venous blood collection |  |  |  |  |  |  | |
|  |  | 5.2.4 Arterial blood collection |  |  |  |  |  |  | |
|  |  | 5.2.5 Sputum aspiration |  |  |  |  |  |  | |
| Proposed additional indicators and their significance: | | | | | | | | |  |

**Part three**

**Instructions for completing the form:**

**Please select the basis for your judgement, level of influence and familiarity with the above entry “√” the appropriate column**

| Basis of judgement | Level of influence | | | | |
| --- | --- | --- | --- | --- | --- |
|  | large | middle | | small | |
| Theoretical analysis |  |  | |  | |
| Practical experience |  |  | |  | |
| References |  |  | |  | |
| Intuitive aspects |  |  | |  | |
| How familiar are you with the content of this survey | | | | | |
| Degree of familiarity | Very familiar | More familiar | General familiar | Not really familiar | Completely unfamiliar |
|  |  |  |  |  |  |

Thank you again for your support and help with this subject.I wish you a happy life. Good luck with your work.
