# Supplementary material for: Construction of a standardized training system for hospital infection prevention and control for new medical staff in internal medicine ICUs based on the Delphi method
Source: PLoS One. 2023 Nov 16;18(11):e0294606. doi: 10.1371/journal.pone.0294606 (PMC10653407; doi:10.1371/journal.pone.0294606)
Supplement: Attachment — Submitted filename: Response to Reviewers.docx [file pone.0294606.s003.docx]

***Point-by-point response to the reviewers’ comments***

Thanks for the reviewers’ comments and consideration for further review for our manuscript. Those comments were all valuable and very helpful for revising and improving our paper. To ensure our manuscript satisfactorily addresses reviewers’ comments, we have improved the main text's clarity in the revised version.

Please let us know if you have any further suggestions. We look forward to hearing from you soon.

**Reviewer #2** (Comments to the Authors):

Comment 1: The authors can join the sentences in line 45 with a conjunction

**Response**: Thanks for your suggestion, we have joined the sentence in line 45 with a conjunction.

In conclusion, the proposed standardized training system for infection prevention and control among new medical staff in the internal medicine ICU is both scientifically sound and practical, which can contribute to improved patient safety, reduced healthcare costs, and enhanced overall quality of care in internal medicine ICUs.

**For details, please see page 3, line 45.**

Comment 2: Avoiding subheadings in the discussion is ideal

**Response**: Thank you very much for your valuable comments. We have removed the subheadings from the discussion.

Comment 3: Line 327 seems to be incomplete

**Response**: Thanks for your reminding, we have completed this sentence.

In addition, it can strengthen patient safety and reduce healthcare-associated infections.

**For details, please see page 27, line 336.**

Comment 4: The section on limitation could be inserted as the last paragraph in the discussion section

**Response**: Thanks for your suggestion, we have moved the section on limitations from the summary to the last part of the discussion.

**For details, please see page 27, lines 321 to 326.**
